# Predicting progression from subjective cognitive decline to mild cognitive impairment or dementia based on brain atrophy patterns

**DOI:** 10.1186/s13195-024-01517-5

**Published:** 2024-07-05

**Authors:** Ondrej Lerch, Daniel Ferreira, Erik Stomrud, Danielle van Westen, Pontus Tideman, Sebastian Palmqvist, Niklas Mattsson-Carlgren, Jakub Hort, Oskar Hansson, Eric Westman

**Affiliations:** 1 Memory Clinic, Department of Neurology, Second Faculty of Medicine, Charles University and Motol University Hospital, Prague, 15006 Czech Republic; 2 Division of Clinical Geriatrics, Centre for Alzheimer Research, Department of Neurobiology, Care Sciences, and Society, Karolinska Institutet, Stockholm, 14183 Sweden; 3 Department of Radiology, Mayo Clinic, Rochester, MN 55902 USA; 4 Clinical Memory Research Unit, Department of Clinical Sciences Malmö, Lund University, Malmö, 20502 Sweden; 5 Memory Clinic, Skåne University Hospital, Malmö, 21428 Sweden; 6 Diagnostic Radiology, Institution for Clinical Sciences Lund, Lund University, Lund, 22184 Sweden; 7 Department of Neuroimaging, Centre for Neuroimaging Sciences, Institute of Psychiatry, Psychology and Neuroscience, King’s College London, London, SE58AF UK

**Keywords:** Structural MRI, Subjective cognitive decline, Alzheimer’s disease, Atrophy patterns, Multivariate analysis

## Abstract

**Background:**

Alzheimer’s disease (AD) is a progressive neurodegenerative disorder where pathophysiological changes begin decades before the onset of clinical symptoms. Analysis of brain atrophy patterns using structural MRI and multivariate data analysis are an effective tool in identifying patients with subjective cognitive decline (SCD) at higher risk of progression to AD dementia. Atrophy patterns obtained from models trained to classify advanced AD versus normal subjects, may not be optimal for subjects at an early stage, like SCD. In this study, we compared the accuracy of the SCD progression prediction using the ‘severity index’ generated using a standard classification model trained on patients with AD dementia versus a new model trained on β-amyloid (Aβ) positive patients with amnestic mild cognitive impairment (aMCI).

**Methods:**

We used structural MRI data of 504 patients from the Swedish BioFINDER-1 study cohort (cognitively normal (CN), Aβ-negative = 220; SCD, Aβ positive and negative = 139; aMCI, Aβ-positive = 106; AD dementia = 39). We applied multivariate data analysis to create two predictive models trained to discriminate CN individuals from either individuals with Aβ positive aMCI or AD dementia. Models were applied to individuals with SCD to classify their atrophy patterns as either high-risk “disease-like” or low-risk “CN-like”. Clinical trajectory and model accuracy were evaluated using 8 years of longitudinal data.

**Results:**

In predicting progression from SCD to MCI or dementia, the standard, dementia-based model, reached 100% specificity but only 10.6% sensitivity, while the new, aMCI-based model, reached 72.3% sensitivity and 60.9% specificity. The aMCI-based model was superior in predicting progression from SCD to MCI or dementia, reaching a higher receiver operating characteristic area under curve (AUC = 0.72; *P* = 0.037) in comparison with the dementia-based model (AUC = 0.57).

**Conclusion:**

When predicting conversion from SCD to MCI or dementia using structural MRI data, prediction models based on individuals with milder levels of atrophy (i.e. aMCI) may offer superior clinical value compared to standard dementia-based models.

## Background

Alzheimer’s disease (AD) is a progressive neurogenerative disease and the most common cause of dementia, with an increasing prevalence worldwide [1]. The pathophysiological changes in AD begin years or even decades before the onset of clinical symptoms [2, 3]. The failure of many recent drug trials suggests that future effective therapeutic strategies may require timely intervention in a preclinical stage [4–6]. To help with identification of individuals with increased risk of AD, the concept of subjective cognitive decline (SCD) has been proposed [7]. Subjective complaints of cognitive decline are a standalone risk factor for the development of mild cognitive impairment (MCI) and dementia with up to twofold risk increase when compared to healthy individuals without complaints [8, 9]. Identification of individuals suffering from SCD due to ongoing neurodegenerative processes such as AD, as opposed to SCD due to other etiology, is a task of substantial clinical importance, because individuals before onset of clinical symptoms are the most likely to benefit from treatment when available [4–6].

Although the current clinical diagnostic algorithm doesn’t recommend routine evaluation of pathophysiological biomarkers in cognitively unimpaired individuals [10], for research purposes, the framework separately evaluating individual biomarkers regardless of clinical syndrome, the “ATN framework”, has been established. In this framework, the “A” stands for a β-amyloid biomarker (e.g. cerebrospinal fluid [CSF] β-amyloid [Aβ] 42 peptide levels, Aβ42/40 ratio, amyloid positron emission tomography [PET]), “T” for a tau biomarker (e.g. CSF P-tau levels, tau PET), and “N” for a neurodegeneration biomarker (e.g. structural MRI, 18 F-fluorodeoxyglucose PET) [11]. In clinical practice, full evaluation of individuals with SCD may prove challenging due to limited availability of biomarkers, ethical and economic considerations. Structural MRI, however, is a widely available, non-invasive, and safe method to assess neuronal damage.

Early stages of AD are typically characterized by a pattern of atrophy with predominant involvement of the medial temporal lobe [12]. A similar atrophy pattern has been observed in SCD individuals [13–18]. Analyzing a specific pattern of atrophy rather than individual structures has been shown to yield high predictive value [12]. We have previously used Orthogonal Projection to Latent Structures (OPLS) [19], a multivariate data analysis method, to discriminate both MCI and patients with AD from controls [12]. We used OPLS to create a “disease severity index”, using multiple structural MRI measures as input, allowing us to predict progression from MCI to dementia [20, 21] and from SCD to MCI or dementia [22].

When the task is to predict progression from MCI to dementia, majority of published studies utilize models based on sets of healthy individuals and patients with AD dementia [23]. However, this approach may have limitations in predicting progression from SCD to MCI. Though some SCD individuals show modest brain atrophy [24], hence they are much closer to healthy individuals than to patients with AD dementia. Such models are therefore more likely to treat SCD individuals with very mild levels of atrophy incorrectly as healthy. To the best of our knowledge, accuracy of prediction using datasets trained on individuals at different stages of the disease (e.g., MCI, AD dementia) has never been compared. We hypothesized, that it may be possible to further improve the prediction accuracy of SCD models, by training the models on individuals with the same pattern but milder levels of atrophy, such as MCI due to AD [25], as opposed to patients with AD dementia. Hence, (1) we used multivariate data analysis and structural MRI data to examine atrophy patterns of β-amyloid positive amnestic MCI patients or patients with AD dementia and β-amyloid negative cognitively normal (CN) individuals, and applied the resulting models to SCD individuals to classify them as CN-like or disease-like; (2) we used the resulting classification as a basis for prediction of progression from SCD to MCI using longitudinal clinical data; and (3) compared the accuracy of prediction of “MCI-based” models with prediction based on equally constructed models based on AD patients with dementia.

## Methods

### Participants

Participants were recruited from the prospective longitudinal Swedish BioFINDER-1 study (NCT01208675) (see http://www.biofinder.se for more information) [26, 27]. A total of 504 individuals were included.

The group of CN participants consisted of 220 β-amyloid negative elderly individuals from the BioFINDER study, which were initially recruited from the population-based Malmö Diet Cancer Study [28]. The inclusion criteria for the CN group were as follows: (1) Age ≥ 60 years; (2) Mini Mental State Examination (MMSE) score in range of 28–30 points [29]; (3) No cognitive symptoms as assessed by a physician with expertise in cognitive disorders; (4) Participant did not fulfill the criteria for either MCI [30] or dementia [31]; (5) was able to speak and understand Swedish in sufficient level not to require an interpreter during the examination; and (6) had normal CSF levels of Aβ42 (> 530pg/ml) [32] at baseline. Exclusion criteria were: (1) Relevant unstable systemic illness or organ failure making it difficult to participate in the study (i.e. terminal cancer, etc.); (2) Relevant neurological or psychiatric illness (major depressive disorder, Parkinson’s disease, stroke, etc.); (3) Current significant alcohol or substance abuse; and (4) Refusal to undergo either MRI or lumbar puncture procedures. Collection of the data took place between 2010 and 2014. In further assessment, we used subgroups of β-amyloid negative CN individuals who were one-to-one age- and sex-matched to the diagnostic group analyzed (i.e. MCI or AD dementia). We used exact matching for sex and loose matching for age, with minimal age difference as a selection criterion.

The group of β-amyloid positive amnestic mild cognitive impairment (aMCI) patients was recruited from the cohort with mild cognitive symptoms of the BioFINDER study and consisted of 106 individuals from the memory clinics at Skåne University Hospital and Ängelholm’s Hospital in Sweden, between 2010 and 2015. All patients had been referred to the memory clinics due to cognitive symptoms experienced by patient or informant, as a part of routine clinical practice. All patients fulfilled the criteria of amnestic MCI - their normative z-score for episodic memory domain in neuropsychological assessment (see next section) was ≤ 1.5. Additional inclusion criteria for the aMCI group were defined as follows: (1) Referral to the memory clinic due to cognitive symptoms (including non-memory complaints); (2) Age between 60 and 80 years; (3) MMSE score of 24–30 points at baseline; (4) Participant did not fulfill the criteria for dementia [31]; (5) Ability to speak and understand Swedish in sufficient level not to require an interpreter during the examination; and (6) abnormal CSF levels of Aβ42 (≤ 530pg/ml) [32] at baseline. MCI patients were classified as amnestic single or multiple domains, based on the results of neuropsychological assessment (see next section) at the baseline. Exclusion criteria for MCI patients were: (1) Relevant unstable systemic illness or organ failure making it difficult to participate; (2) Current significant alcohol or substance abuse; (3) Refusal to undergo either lumbar puncture or neuropsychological assessment; and (4) Cognitive symptoms at baseline explainable by another condition (normal pressure hydrocephalus, brain tumor, major stroke, epilepsy, schizophrenia, past significant alcohol abuse and ongoing medication such as benzodiazepines).

The group of patients with SCD was recruited from the cohort with mild cognitive symptoms of the BioFINDER study and consisted of 139 individuals included between 2010 and 2015 from the memory clinics at Skåne University Hospital and Ängelholm’s Hospital in Sweden. As in the MCI group, all patients had been referred to the memory clinics due to cognitive symptoms experienced by patient or informant, as a part of routine clinical practice. No further specific questionnaires to ascertain SCD were administered. Inclusion criteria were similar to the MCI group criteria 1–5. However, SCD individuals showed no objective impairment in neuropsychological testing based on established normative data. Exclusion criteria were equal to those of the MCI group.

The group of patients with dementia was recruited from the dementia cohort of the BioFINDER study and consisted of 39 individuals included between 2010 and 2015. Patients were diagnosed with dementia after thorough clinical investigation at the memory clinic from the Skåne University Hospital. All patients fulfilled the criteria of probable dementia due to AD [33], fulfilling at minimum the core clinical criteria. Most AD patients, though not all (*n* = 32; 82.05%), underwent lumbar puncture and had CSF evidence of abnormal levels of Aβ42 (≤ 530pg/ml). The exclusion criteria were defined as (1) significant unstable systemic illness or organ failure such as terminal cancer, making it difficult to participate in the study; or (2) current significant alcohol or substance misuse.

### Neuropsychological assessment

All participants underwent neuropsychological evaluation, which consisted of tests assessing verbal, visuospatial and construction skills, episodic memory, and executive functions. Individual test batteries varied between groups. Tests administered to all groups included measures of global cognition – MMSE and AD Assessment Scale-Cognitive subscale (ADAS-cog) [34]. Global deterioration scale [35] was used as an outcome measure in further analyses. For further details, please see http://biofinder.se/data-biomarkers/clinical-evaluation/.

### CSF sampling

The CSF analysis was performed in all participants in accordance with the Alzheimer’s Association Flow Chart for CSF biomarkers [36]. The samples were collected at baseline and stored in 1mL polypropylene tubes at temperature of -80 °C. The CSF levels of Aβ42 were analyzed simultaneously in a single laboratory with the INNOTEST ELISA set (Fujirebio Europe, Ghent, Belgium) [37].

### MRI acquisition

All participants underwent magnetic resonance imaging (MRI) scanning using a 3T Siemens Trio Tim scanner (Munich, Germany) at Skåne University Hospital, Sweden. The imaging protocol included a high resolution T1-weighted scan acquired with a magnetization-prepared rapid acquisition gradient echo sequence (176 slices; repetition time = 1950–2000 ms; echo time = 3.37 ms; inversion time = 900 ms; flip angle = 9°; voxel size = 0.97 * 0.97 * 1.2mm^3^).

### MRI analysis

The acquired T1 images were analyzed using the FreeSurfer 6.0 imaging suite (https://surfer.nmr.mgh.harvard.edu/) with the in-house database system theHiveDB [38]. For each individual, the thickness of 34 cortical regions [39] and the volumes of 23 subcortical structures [40] were obtained from FreeSurfer. All segmentations were visually checked prior to further processing, only the subjects that passed the visual inspection were included in subsequent analyses. The summary measures of CSF, white and grey matter volumes were not included in the model to avoid redundancy, as well as volume of brainstem and cerebellum, as these regions undergo minimal levels of atrophy in the early stages of the disease [41]. Left and right-sided measures were averaged prior to analysis. We performed principal component analysis on these 34 + 17 measures within each study group (CN, aMCI, AD dementia), to detect possible outliers. We found no individuals with scores larger than 4 SD in first or second component within their respective group, indicating that this dataset did not have any outliers.

### Statistical methods

#### Participants

We used the R software (R Foundation for Statistical Computing, Vienna, Austria; www.r-project.org) to perform the statistical analyses. We used analysis of variance (ANOVA) to assess group differences in age and analysis of covariance (ANCOVA) using age and sex as covariates to assess differences in education, neuropsychological test results, MRI and CSF measurements. The Kruskal-Wallis test was used to assess the differences in sex and *APOE* ε4 distributions. For groups characterization, to reduce the number of reported volumetric measurements, we reported volumes or thickness of selected regions known to be affected in the earliest stages of AD (i.e. hippocampus, entorhinal cortex) according to Braak and Braak [42]. We performed 2 separate ANOVA and ANCOVA analyses: First, for groups associated with AD dementia (SCD, AD dementia, matched β-amyloid negative CN), second, for groups associated with aMCI (SCD, β-amyloid positive aMCI, matched β-amyloid negative CN).

#### Training of the OPLS model

To calculate the “severity index” [22] that assesses the pattern of atrophy characteristic of patients with AD dementia (or aMCI) versus controls, we employed the OPLS [19] algorithm using the “ropls” package implemented within the R-programming environment (https://bioconductor.org/packages/release/bioc/html/ropls.html). The implementation used original non-linear iterative partial least squares (NIPALS) [43] algorithms [19, 44]. The OPLS has been previously extensively used in CN vs. AD classification and SCD to MCI progression prediction [20, 22, 45–50] and its performance has been shown be similar to that of other commonly used multivariate analysis algorithms [50]. The procedure for the actual index has been described in detail previously [20, 45]. In brief, the data is preprocessed using standard steps, applying unit variance scaling and mean centering to the data. The OPLS algorithm then splits the systemic variation into two parts - predictive and orthogonal. The first, predictive component, contains information relevant for the classification between CN and aMCI/dementia groups. The second, orthogonal component, contains information that is not related to the classification problem. The ability to predict and the reliability of the model are evaluated through the ‘goodness of fit’ or explained variance (*R*^*2*^) and the ‘goodness of prediction’ or predicted variance (*Q*^*2*^) parameters. *Q*^*2*^ represents a performance of the model outside of the training dataset and is therefore regarded as a more relevant metric. A value of *Q*^*2*^ > 0.05 is regarded as significant, and a value > 0.5 represents a good model [51]. We used a 10-fold cross-validation [52] for training of the model.

**Fig. 1:**
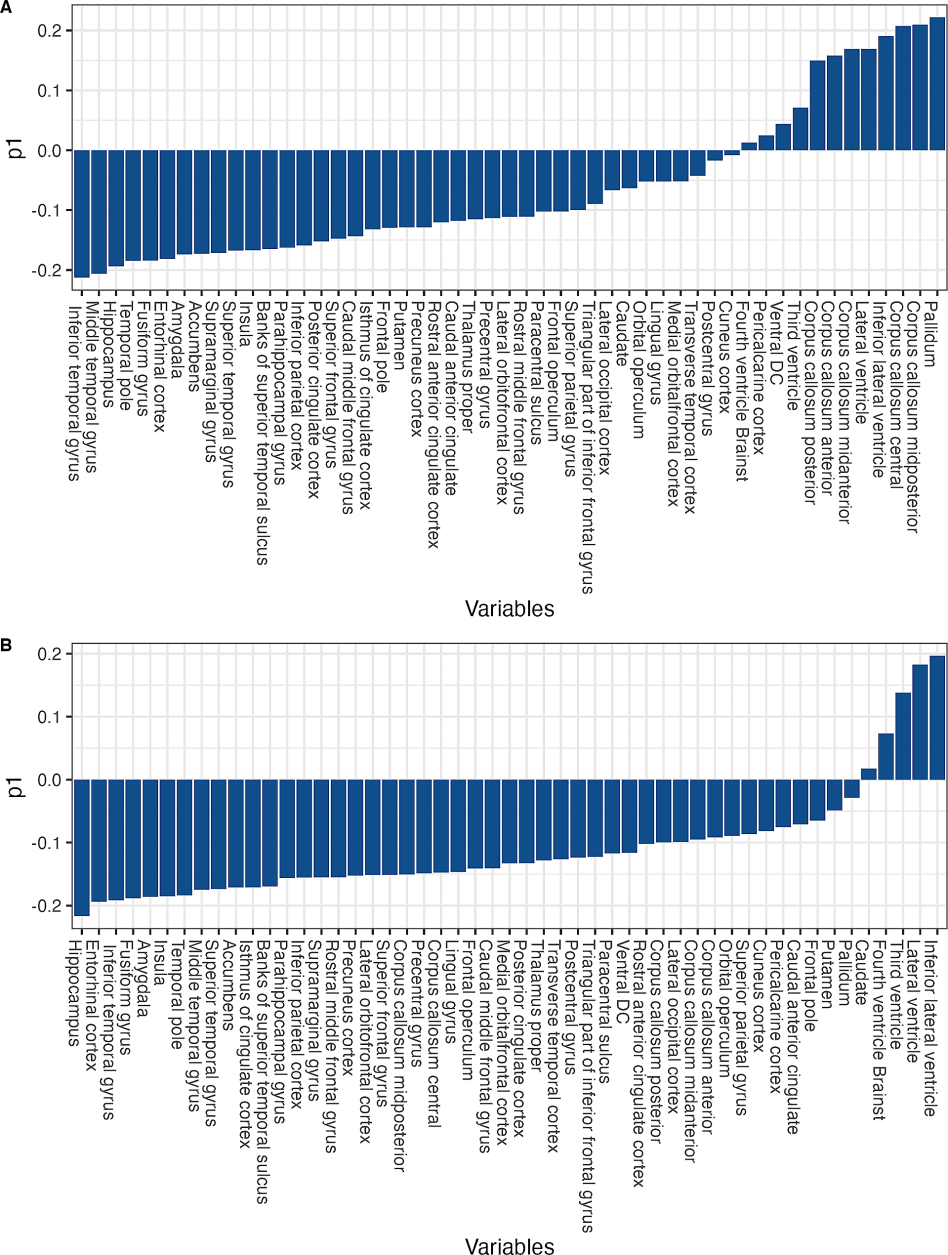
Variable loadings. *p1* = Contribution of individual variables to the predictive component in the model (**A**) trained on the Alzheimer’s disease dementia patients (**B**) trained on the aMCI patients

We used a total of 51 variables from the baseline MRI FreeSurfer assessment as the input data, including the 34 cortical and 17 subcortical regions explained above (Fig. 1A, B). Prior to the analysis, all subcortical volumes were adjusted for the differences in head size by regressing out the estimated total intracranial volume (eTIV) [53, 54]. In addition, we applied a linear detrending algorithm based on age-related changes in the β-amyloid negative CN group to the data, assuming that thickness/volumetric changes in the CN group are mostly associated with aging, while changes in the aMCI and AD dementia groups may also be influenced by disease-related factors. This approach has shown to have a positive effect on the classification performance of OPLS models [49]. For training of the OPLS model, participants from the CN group were assigned a value of 0, while aMCI and AD dementia individuals were assigned a value of 1 during training of their respective models.

In all MRI-based models, prediction accuracy of the model is limited by the heterogeneity of the underlying pathology. In AD, several different pathology phenotypes have been described [55], with correspondingly different atrophy patterns [56–58] including the minimal atrophy phenotype [58]. To minimize the impact of heterogeneity on prediction accuracy of our model, we removed aMCI and demented individuals with the minimal atrophy phenotype [56, 58, 59] from their respective training dataset. Patients in this phenotype are known to have no or low levels of brain atrophy, which may introduce noise in our OPLS classification models. Since the OPLS approach is based on analyzing atrophy patterns, we hypothesized that removal of these individuals from the training dataset would improve the accuracy of the resulting model further. To identify individuals with a minimal atrophy phenotype, we projected all patients from the aMCI and AD dementia group onto their respective models (CN vs. aMCI, and CN vs. AD dementia, respectively), assigning them the predicted value of the “severity index” and classifying them as either CN-like or disease-like. For this classification we used the cutoff value obtained by identifying the point of maximum separation between the smoothed cumulative distribution function of the two groups (i.e., CN and aMCI or CN and AD dementia) [60]. This way we identified 15 individuals from the aMCI group, classified as CN-like, showing minimal atrophy. These individuals were removed from the training dataset. We found no individuals with minimum atrophy in the AD dementia group. Hence, we then repeated the previously described procedures only for the aMCI group, and the model was retrained using an updated training set. The updated set for the aMCI-based model without minimal atrophy patients included 91 aMCI patients. The dementia-based model remained unchanged, including 39 AD dementia patients. For each model, we selected a subgroup of age- and sex-matched β-amyloid negative CN individuals. We used exact matching for sex and loose matching for age, with minimal age difference as a selection criterion. We used the cross-validated model to estimate *Q*^*2*^ and *R*^*2*^ and report sensitivity and specificity values. For more details on how removal of minimal atrophy group affected model performance, see the results.

In total, we built two models, (1) “dementia-based” model, trained using β-amyloid negative CN and AD dementia individuals; and (2) “aMCI-based” model, trained using β-amyloid negative CN and β-amyloid positive aMCI, excluding those with minimal atrophy phenotype. These two models did not differ in any other parameter.

#### Classification

We projected all participants from the SCD group (*n* = 139), regardless of their Aβ status, onto the models (1) and (2), and their values of *Y* or “severity index” for each model were estimated. The cutoff value for predicting observations as either CN-like or disease-like was obtained by identifying the point of maximum separation between the smoothed cumulative distribution function of the two groups (i.e., CN and aMCI or CN and AD dementia), as described above. The final cutoff values used were 0.413 for the dementia-based model and 0.384 for the aMCI-based model.

#### Longitudinal analysis

Next, we assessed the longitudinal clinical data of the SCD individuals over an 8-years follow-up period with regard to their clinical trajectory. We defined clinical trajectory as the progression from SCD to MCI or dementia using the Global deterioration scale. Participants who scored ≥ 3 during the yearly evaluation were treated as progressors. SCD participants were followed up until progression to MCI or dementia or censored on the last date observed. We did not have mortality data available. Longitudinal data were then used to assess sensitivity and specificity of the OPLS models to predict progression. We also used the calculated “severity index” value to compute receiver operating characteristic (*ROC*) and area under curves (*AUC*). Further, we evaluated the clinical trajectory of CN-like and disease-like SCD groups, by performing survival analysis using Kaplan-Meier estimate and log rank test and estimated the risk of progression to MCI or dementia by applying data to the Cox models. Then, we made a comparison of the *ROC* curves of models (1) and (2) using the implementation of DeLong algorithm [61] within the pROC package [62].

**Fig. 2:**
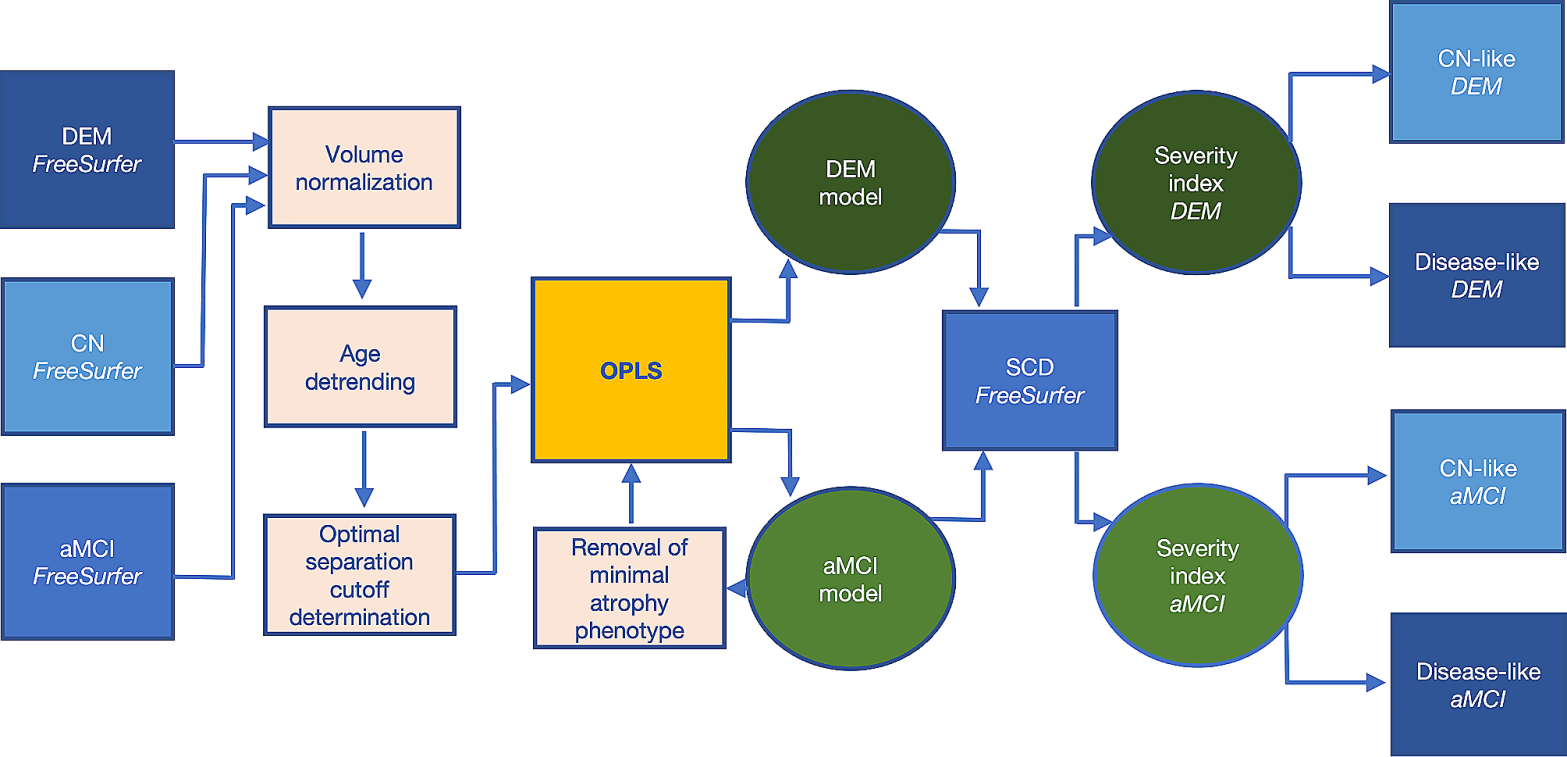
Simplified overview of data-processing steps. Processing preceding computation of the “disease severity index” and prediction of progression; aMCI = β-amyloid positive amnestic mild cognitive impairment; CN = β-amyloid negative cognitively normal participants; DEM = dementia due to Alzheimer’s disease; OPLS = Orthogonal Projection to Latent Structures; SCD = subjective cognitive decline; Individual steps are described in detail in the manuscript

Finally, we compared the models (1) “dementia-based” and (2) “aMCI-based”, regarding their sensitivity, specificity, and *ROC AUC*, as well as in terms of characteristics of SCD groups identified as “disease-like” by each model. Simplified overview of the data processing steps is available in Fig. 2.

## Results

Participant’s main demographical and clinical characteristics are summarized in Table 1. The AD dementia associated groups (SCD, AD dementia, matched β-amyloid negative CN) differed in cognitive performance, *APOE* ε4 allele frequency, volumetric measures, and CSF biomarkers. The aMCI associated groups (SCD, β-amyloid positive aMCI, matched β-amyloid negative CN) differed in age, cognitive performance, *APOE* ε4 allele frequency, volumetric measures, and CSF biomarkers.

**Table 1:** Participant characteristics

	SCD	CN(-)_aMCI_	aMCI(+)	CN(-)_DEM_	DEM	Total	*P*_aMCI_	*P*_DEM_
*N*	139	91	91	39	39	399	-	-
Female (%)	43.17%	51.65%	51.65%	35.9%	35.9%	45.61%	0.322^a^	0.580^a^
Age	70.05 (5.71)	72.28 (4.24)	72.11 (4.62)	71.08 (5.77)	68.49 (8.41)	70.98 (5.63)	0.001**^b^	0.185^b^
Education (years)	12.68 (3.43)	12.04 (3.24)	11.58 (3.44)	13.69 (3.95)	11.05 (3.35)	12.25 (3.49)	0.598^c^	0.110^c^
MMSE	28.76 (1.30)	29.19 (0.94)	26.79 (1.76)	29.49 (0.68)	20.24 (3.82)	27.69 (3.08)	< 0.001***^c^	< 0.001***^c^
ADAS 10 word delayed recall	2.84 (1.74)	1.71 (1.71)	7.33 (1.94)	1.33 (1.22)	8.06 (2.08)	3.68 (2.97)	< 0.001***^c^	< 0.001***^c^
*APOE* ε4 (%)	41.73%	19.78%	73.63%	15.38%	71.79%	44.36%	< 0.001***^a^	< 0.001***^a^
Mean hippocampal volume (mm^3^)^+^	3706.10 (561.00)	3852.79 (431.44)	3068.25 (419.99)	3821.80 (412.79)	3017.96 (427.91)	3538.13 (585.00)	< 0.001***^c^	< 0.001***^c^
Mean entorhinal cortex thickness (mm)^+^	2.90 (0.49)	2.95 (0.46)	2.37 (0.38)	2.97 (0.41)	2.45 (0.30)	2.75 (0.50)	< 0.001***^c^	0.002**^c^
Total intracranial volume (cm^3^)	1563.43 (144.64)	1575.07 (153.04)	1569.24 (138.51)	1561.21 (138.43)	1551.29 (173.36)	1566.00 (147.09)	0.154^c^	0.396^c^
Aβ42 positivity (%)	31.65%	0%	100%	0%	78.12%	40.82%	< 0.001***^a^	< 0.001***^a^
Aβ42 level (pg/ml)	658.16 (213.88)	747.04 (132.72)	377.48 (89.09)	746.03 (136.70)	427.22 (183.95)	603.53 (221.00)	< 0.001***^c^	< 0.001***^c^
Tau level (pg/ml)	322.13 (135.86)	283.98 (71.86)	459.76 (190.17)	286.64 (81.00)	762.84 (278.25)	377.67 (201.20)	< 0.001***^c^	< 0.001***^c^
P-tau level (pg/ml)	54.97 (24.16)	50.80 (12.88)	74.05 (31.51)	51.85 (14.04)	103.06 (67.89)	62.05 (32.96)	< 0.001***^c^	< 0.001***^c^

Values are expressed as: mean (standard deviation) unless indicated otherwise; *: *P* < 0.05; **: *P* < 0.01; ***: *P* < 0.001; ^+^Selected volumetric measures based on their early involvement during Alzheimer’s disease onset according to Braak & Braak, 1991; ^a^Kruskal-Wallis test; ^b^ANOVA (analysis of variance); ^c^ANCOVA (analysis of covariance; covariates: sex, age); SCD = subjective cognitive decline; aMCI(+)= β-amyloid positive amnestic mild cognitive impairment; CN(-)_aMCI_= β-amyloid negative cognitively normal participants age- and sex-matched to the aMCI group; DEM = dementia due to Alzheimer’s disease; CN(-)_DEM_= β-amyloid negative cognitively normal participants age- and sex-matched to the AD dementia group; *P*_aMCI_ = p-value of the analysis performed on groups associated with amnestic mild cognitive impairment (SCD, CN(-)_aMCI_, aMCI(+)); *P*_DEM_ = p-value of the analysis performed on groups associated with dementia due to Alzheimer’s disease (SCD, CN(-)_DEM_, DEM); MMSE = Mini-Mental State Examination, ADAS = Alzheimer’s Disease Assessment Scale; Aβ42 positivity: Percentage of individuals with CSF level of β-amyloid 42 peptide lower then 530pg/ml; Aβ42 level: CSF levels of β-amyloid 42 peptide in pg/ml; Tau level: CSF levels of tau protein in pg/ml; P-tau level: CSF levels of phosphorylated tau protein in pg/ml

### Classification results

#### Classification using Alzheimer’s disease dementia patients (standard approach)

**Fig. 3:**
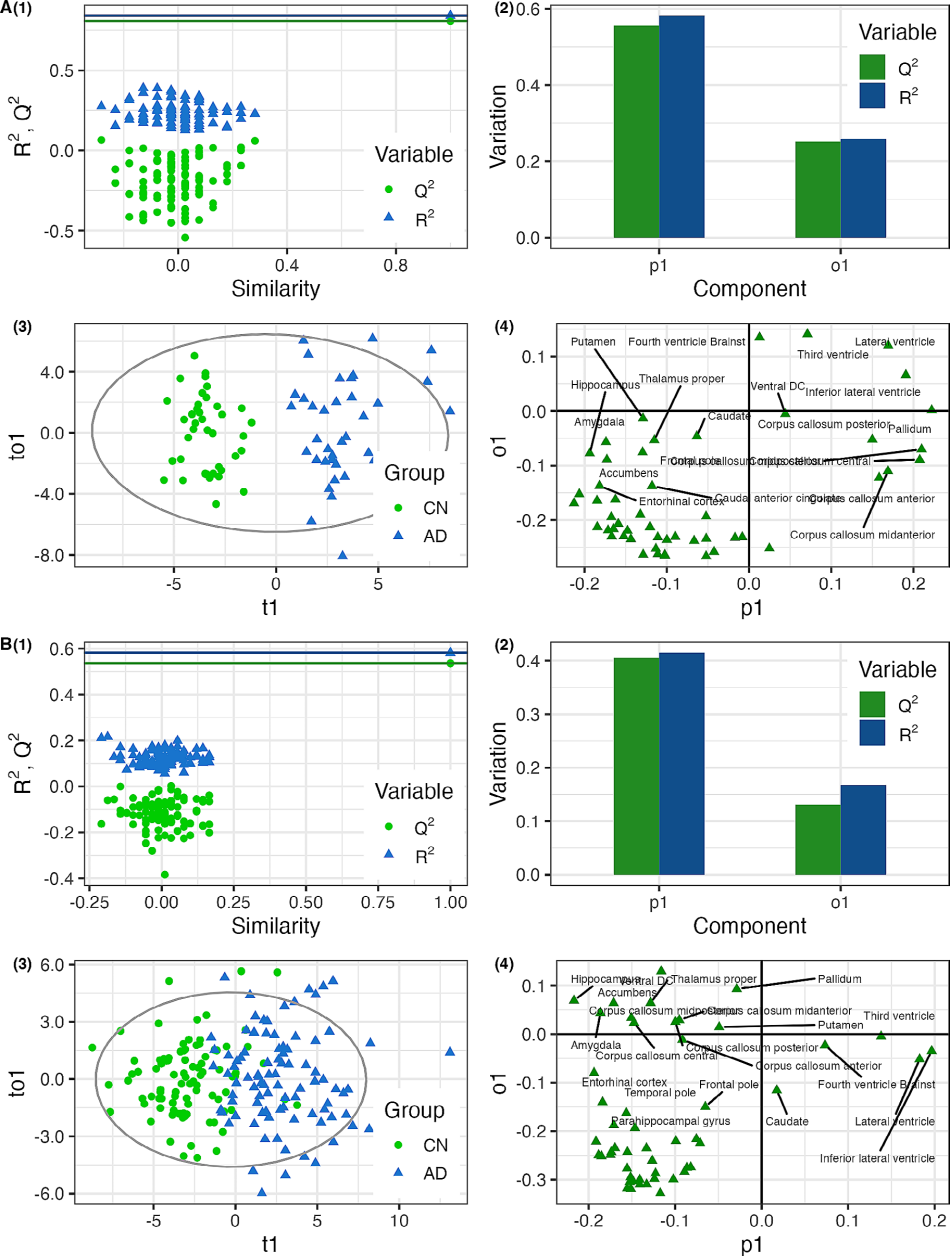
Characteristics of the model. (**A**) trained on the Alzheimer’s disease dementia patients (**B**) trained on the aMCI patients ; *R*^*2*^ = explained variance; *Q*^*2*^ = predicted variance; (1) Permutation plot: Comparison of *R*^*2*^ and *Q*^*2*^ values of the model with other models, where random permutations of *Y* (diagnostic information) have been performed while *X*-data (input data) stayed intact; (2) *Q*^*2*^ and *R*^*2*^ values of individual components: *p1* = predictive component; *o1* = first orthogonal component (3) Score plot: individual scores of participants used in training; *t1* = predictive component score; *to1* = first orthogonal component score; (4) Loading plot: loadings of individual variables; *p1* = predictive component; *o1* = first orthogonal component

The cross-validated “AD-dementia-based” model reached a cumulative *R*^*2*^ of 0.842 and a cumulative *Q*^*2*^ of 0.807. The model reached 100% sensitivity and 100% specificity in discriminating patients with AD dementia from CN individuals. Detailed model characteristics are summarized in Figs. 1A and 3A. Removal of patients with minimal atrophy did not affect this model since no patients were removed. When applied to the SCD data, the model labelled 96.4% of the SCD individuals as CN-like (*n* = 134; 31.3% β-amyloid positive) and 3.6% of the SCD individuals as AD dementia-like (*n* = 5; 40.0% β-amyloid positive). The AD dementia-like SCD group was older and had lower hippocampal volume than the CN-like SCD group after correcting for age and sex (*P* < 0.05). It did not differ from the CN-like SCD group in other characteristics (Table 2).

**Table 2:** Classification of SCD individuals

		AD dementia-based model				aMCI-based model		
		CN-like	AD-like	*P*		CN-like	aMCI-like	*P*
*N*		134	5	-		69	70	-
Female (%)		42.54%	60%	0.441^a^		44.93%	41.43%	0.678^a^
Age		69.85 (5.62)	75.54 (5.70)	0.028*^b^		69.16 (5.39)	70.93 (5.91)	0.068^b^
Education (years)		12.74 (3.40)	11.00 (4.18)	0.702^c^		13.20 (3.68)	12.16 (3.10)	0.415^c^
Severity index		0.09 (0.14)	0.47 (0.04)	0.003**^c^		0.16 (0.15)	0.62 (0.22)	< 0.001***^c^
MMSE		28.77 (1.29)	28.60 (1.67)	0.616^c^		28.91 (1.05)	28.61 (1.50)	0.987^c^
ADAS 10 word delayed recall		2.74 (1.66)	5.60 (1.52)	0.076^c^		2.57 (1.58)	3.12 (1.86)	0.869^c^
*APOE* ε4 (%)		40.3%	80%	0.172^a^		40.58%	42.86%	0.563^a^
Mean hippocampal volume (mm^3^)^+^		3597.84 (404.50)	2377.45 (672.87)	0.004**^c^		3822.68 (307.98)	3289.04 (456.93)	< 0.001***^c^
Mean entorhinal cortex thickness (mm)^+^		2.76 (0.41)	2.09 (0.50)	0.082^c^		2.93 (0.35)	2.54 (0.41)	< 0.001***^c^
Total intracranial volume (cm^3^)		1559.60 (144.53)	1666.09 (116.29)	0.563^c^		1561.44 (131.97)	1565.38 (157.06)	0.818^c^
Aβ42 positivity (%)		31.34%	40%	0.684^a^		26.09%	37.14%	0.163^a^
Aβ42 level (pg/ml)		660.79 (214.67)	587.60 (198.77)	0.254^c^		668.42 (217.60)	648.04 (211.23)	0.805^c^
Tau level (pg/ml)		320.65 (134.10)	361.80 (192.08)	0.685^c^		336.64 (144.19)	307.83 (126.53)	0.612^c^
P-tau level (pg/ml)		54.57 (23.86)	65.80 (32.68)	0.542^c^		57.96 (26.04)	52.03 (21.94)	0.609^c^

Values are expressed as: mean (standard deviation) unless indicated otherwise; *: *P* < 0.05; **: *P* < 0.01; ***: *P* < 0.001; ^+^Selected volumetric measures based on their early involvement during Alzheimer’s disease onset according to Braak & Braak, 1991; ^a^Kruskal-Wallis test; ^b^ANOVA (analysis of variance); ^c^ANCOVA (analysis of covariance; covariates: sex, age); CN-like = individuals classified as cognitively-normal-like, AD-like = individuals classified as Alzheimer’s disease dementia-like; aMCI-like = individuals classified as amnestic mild cognitive impairment-like; MMSE = Mini-Mental State Examination, ADAS = Alzheimer’s Disease Assessment Scale; Aβ-42 positivity: Percentage of individuals with CSF level of β-amyloid 42 peptide lower then 530pg/ml ; Aβ-42 level: CSF levels of β-amyloid 42 peptide in pg/ml; Tau level: CSF levels of tau protein in pg/ml; P-tau level: CSF levels of phosphorylated tau protein in pg/ml;

#### Classification using aMCI patients (new approach)

The cross-validated “aMCI-based” model reached a cumulative *R*^*2*^ of 0.582 and a cumulative *Q*^*2*^ of 0.536. The model reached 96.7% sensitivity and 80.2% specificity in discriminating patients with aMCI from CN individuals. More detailed model information is summarized in Figs. 1B and 3B. Initial model, without removal of patients with minimal atrophy phenotype, reached lower cross validated sensitivity (87.74%), while having only marginally higher specificity (82.08%). This model also showed worse performance when applied to external dataset during cross validation (*Q*^*2*^ = 0.425) and was therefore considered loss robust.

Further, to evaluate the effect of training set size on model performance - since both models were trained using different number of patients (39 vs. 91) - we retrained the aMCI-based model, using a subset of 39 randomly selected individuals from the aMCI dataset, keeping all other parameters identical. The resulting aMCI model was significant (*Q*^2^ = 0.563), showing lower sensitivity (59.57% vs. 72.34%) but higher specificity (73.91% vs. 60.87%) and similar *ROC AUC* (0.719 vs. 0.72) when predicting progression from SCD to MCI and dementia (see next section), compared to the model trained on full number of participants. Comparing *ROC* curves, it didn’t perform differently from the full model (*p* = 0.998). In further analyses, we only evaluated model trained on full number of participants, excluding patients with minimal atrophy phenotype.

Applying the model to the SCD data, 49.6% of individuals (*n* = 69; 26.1% β-amyloid positive) were labelled as CN-like and 50.4% (*n* = 70; 37.1% β-amyloid positive) as aMCI-like. The aMCI-like SCD group had lower hippocampal volume and thinner entorhinal cortex than the CN-like SCD group after correcting for sex and age (*P* < 0.05). The aMCI-like SCD group did not differ from the CN-like SCD group in other characteristics. (Table 2)

#### Longitudinal analysis

Next, we analyzed the longitudinal data of the 139 SCD participants collected within the 8 years period. Within this period, 47 patients (33.81%) progressed to MCI or dementia, while 92 (66.19%) remained in the SCD group. Most participants progressed within the first 1–2 years after baseline (*n* = 35, 74.4%), and no SCD individual progressed later than the 6th year. SCD progressors were older, had a higher percentage of *APOE* ε4 carriers and a higher percentage of Aβ42 positive individuals (*P* < 0.01). After correcting for sex and age, they scored higher in severity index, performed worse in ADAS 10 word delayed recall but not MMSE at baseline and had lower baseline hippocampal volume (*P* < 0.05). SCD progressors also had lower CSF Tau (*P* = 0.026) but not Aβ42 or P-tau levels at baseline (Table 3).

**Table 3:** SCD progressors versus SCD non-progressors within the 8 years follow-up period

	Progressors	Non-progressors	*P*
*N*	47	92	-
Female (%)	53.19%	38.04%	0.089^a^
Age	72.55 (5.10)	68.77 (5.60)	< 0.001***^b^
Education (years)	11.68 (3.48)	13.18 (3.30)	0.090^c^
Severity index	0.54 (0.31)	0.32 (0.27)	0.048*^c^
MMSE	28.45 (1.47)	28.92 (1.18)	0.226^c^
ADAS 10 word delayed recall	3.80 (1.82)	2.36 (1.49)	0.036*^c^
*APOE* ε4 (%)	55.32%	34.78%	0.002**^a^
Mean hippocampal volume (mm^3^)^+^	3328.02 (564.48)	3669.36 (370.18)	0.034*^c^
Mean entorhinal cortex thickness (mm)^+^	2.60 (0.47)	2.81 (0.39)	0.175^c^
Total intracranial volume (cm^3^)	1571.20 (166.54)	1559.46 (132.88)	0.104^c^
Aβ42 positivity (%)	55.32%	19.57%	< 0.001***^a^
Aβ42 level (pg/ml)	558.23 (226.20)	709.21 (188.98)	0.091^c^
Tau level (pg/ml)	392.94 (164.38)	285.96 (102.09)	0.026*^c^
P-tau level (pg/ml)	66.36 (29.42)	49.15 (18.62)	0.096^c^

Values are expressed as: mean (standard deviation) unless indicated otherwise; *: *P* < 0.05; **: *P* < 0.01; ***: *P* < 0.001; ^+^ Selected volumetric measures based on their early involvement during Alzheimer’s disease onset according to Braak & Braak, 1991; ^a^Kruskal-Wallis test; ^b^ANOVA (analysis of variance); ^c^ANCOVA (analysis of covariance; covariates: sex, age); MMSE = Mini-Mental State Examination, ADAS = Alzheimer’s Disease Assessment Scale; Aβ-42 positivity: Percentage of individuals with CSF level of β-amyloid 42 peptide lower then 530pg/ml ; Aβ-42 level: CSF levels of β-amyloid 42 peptide in pg/ml; Tau level: CSF levels of tau protein in pg/ml; P-tau level: CSF levels of phosphorylated tau protein in pg/ml;

#### Longitudinal analysis using the Alzheimer’s disease dementia model

**Fig. 4:**
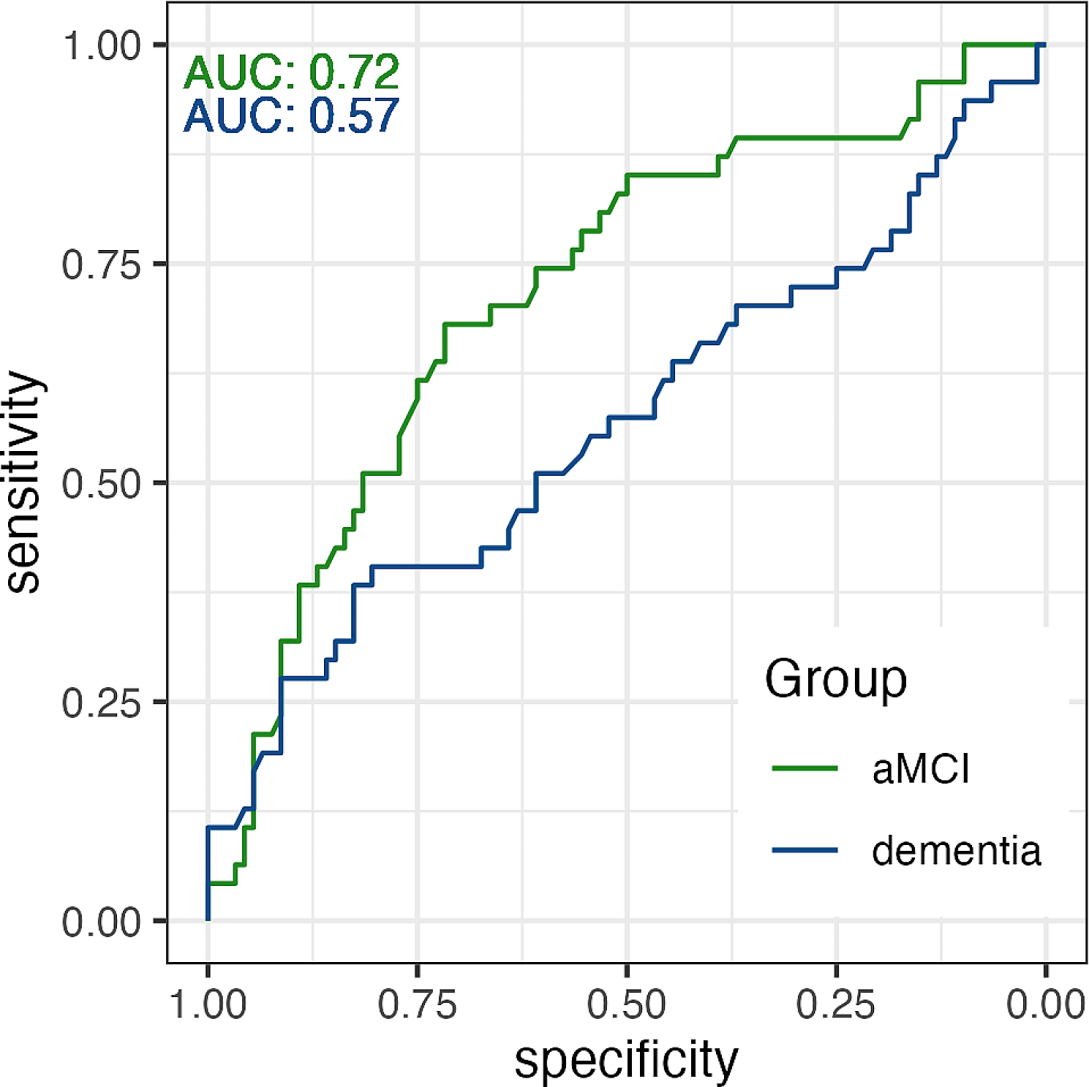
Receiver operating characteristic curves. Curves of the ‘disease severity index’ generated using aMCI-based (green) and dementia-based (blue) models; *AUC* = area under curve

**Fig. 5:**
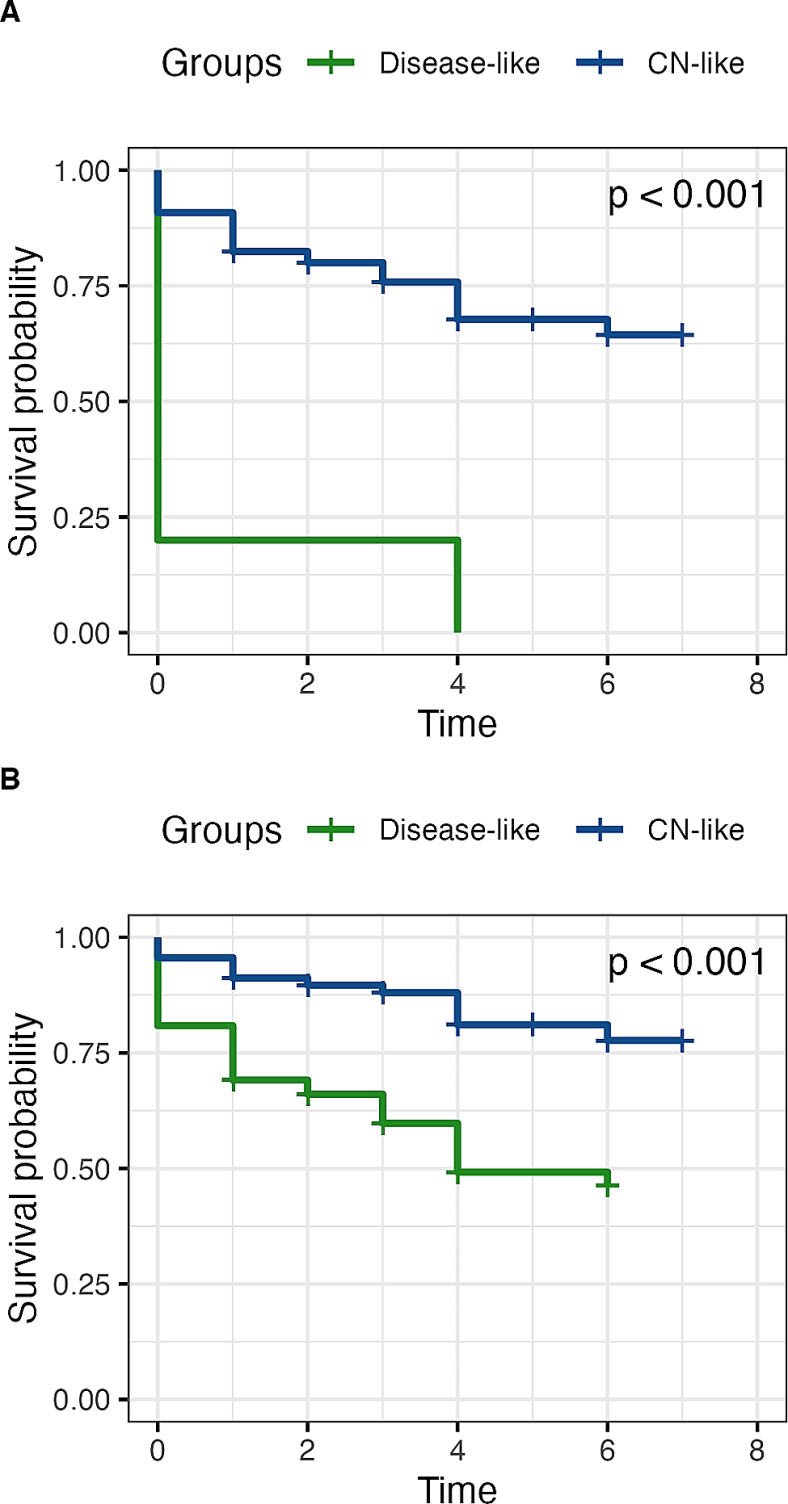
Longitudinal progression of SCD groups. (**A**) using the model based on Alzheimer’s disease dementia patients (**B**) using the model based on aMCI patients; The survival event was defined by either progressing to MCI or dementia at the time of annual follow-up. The log rank test was used to test the difference between the curves

100% of the SCD participants (*n* = 5) labelled as AD dementia-like using the “dementia-based” model progressed to MCI or dementia. This represented 10.6% of all progressors since 42 SCD participants classified as CN-like (31%) also progressed to MCI or dementia. Therefore, the dementia-based model reached 100% specificity but only 10.6% sensitivity in predicting progression from SCD to MCI or dementia in our dataset, resulting in *AUC* of 0.57 (Fig. 4). In the survival analysis using the Kaplan-Meier estimator and log rank test, we found that AD dementia-like SCD participants were more likely to progress to MCI or dementia (*P* < 0.001) than CN-like SCD participants (Fig. 5A). Fitting the data into the Cox-model, we found that AD dementia-like SCD participants were 10.8 times more likely to progress to MCI or dementia than CN-like SCD participants (confidence interval [*CI*]: 4.0–28.9; *P* < 0.001). β-amyloid positivity increased the risk of clinical progression to MCI or dementia 4.3 times (*CI*: 2.4–7.9; *P* < 0.001), while sex did not affect the risk of progression (*P* = 0.679).

#### Longitudinal analysis using the aMCI model

Out of the 70 SCD patients labelled as aMCI-like using the “aMCI-based” model, 48.6% (*n* = 34) progressed to MCI or dementia. The model thus identified correctly 72.3% of all SCD progressors. Out of the CN-like group, only 18.8% (*n* = 13) progressed to MCI. Therefore, the aMCI model reached 72.3% sensitivity and 60.9% specificity in predicting progression from SCD to MCI and dementia. The *AUC* reached a value of 0.72 (Fig. 4). Performing the survival analysis using the Kaplan-Meier estimator and log rank test, we found that aMCI-like SCD participants were more likely to progress to MCI or dementia (*P* < 0.001) than CN-like SCD participants (Fig. 5B). Fitting the data into the Cox-model, we found that aMCI-like SCD participants were 2.9 times more likely to progress to MCI or dementia than CN-like SCD participants (*CI*: 1.5–5.6; *P* = 0.001). β-Amyloid positivity increased the risk to progress to MCI or dementia 3.4 times (*CI*: 1.8–6.4; *P* < 0.001). Sex did not affect the risk of progression (*P* = 0.406).

#### ROC comparison

Comparing the *ROC* curves, we found that the models performed differently between groups (*P* = 0.037) (Fig. 4). The AD dementia-based model identified a lower number of individuals (*n* = 5) at high risk of progression, most of which progressed by the first follow up visit, and all of whom progressed within first four years. The aMCI-based model identified a larger group of individuals (*n* = 70) with moderate risk of progression, progressing in up to 6 years after the initial scan. The aMCI-based model achieved higher *ROC AUC* than the dementia-based model (0.72 vs. 0.57, respectively).

## Discussion

In this study, we used multivariate data analysis and structural MRI to compare classification and prediction models for SCD. We assessed the frequency of disease-like SCD individuals and their characteristics in comparison with CN-like SCD individuals and evaluated the accuracy of prediction of progression from SCD to MCI or dementia, using equally constructed models based on either β-amyloid positive aMCI or AD dementia patient data.

Comparing the dementia-based and the aMCI-based models, the dementia-based model achieved higher values of explained variance (*R*^*2*^) and goodness of prediction (*Q*^*2*^) metrics as well as better overall cross-validated sensitivity and specificity (100% and 100%, respectively) than the aMCI-based model (96.7% and 80.2%, respectively). This was expected, since overall levels of atrophy in AD dementia are higher than in aMCI [63], supposedly making classification of aMCI vs. CN individuals based on atrophy patterns more difficult than the classification of AD dementia vs. CN. This corresponds to our previous results on an external cohort [12], where the dementia-based model also reached higher cross-validated sensitivity and specificity values than the MCI-based model (81% vs. 66% and 82% vs.73%, respectively). Other previous works using the OPLS [20–22] based their models on AD dementia patients only, reaching cross validated sensitivity between 84 and 87% and specificity between 90 and 100%. Both our models therefore reached higher sensitivity and specificity values than similarly built models in the previous studies [12, 20–22]. Part of this improvement may be explained by factors such as smaller size of the AD dementia training dataset (*n* = 39) or overall homogeneity of our dataset (all participants come from a single center, MRI scans were performed using the same scanner) leading to a slight overfitting. However, we believe other factors to be of more importance. Unlike the previous studies, we used training datasets based on biomarker defined individuals – β-amyloid positive aMCI and AD dementia patients with age and sex matched β-amyloid negative CN individuals. We have also introduced several methodological improvements into the model creation, most importantly removal of individuals with minimal atrophy phenotype from the training dataset, which has led to a notable improvement of the aMCI-based model. Further methodological improvements included identification of optimal cutoff value and age detrending. This contributes to the novelty of the current study, but also provided high sensitivity and specificity values for the MCI vs. CN classification (96.7% and 80.2%), which are usually around 75–85% in the literature [12, 64–66], though some authors report both sensitivity and specificity as high as 100%, using a combination of multiple MRI-based features [67].

Looking at the individual variable loadings, among the most important variables contributing to the dementia-based model were thickness of inferior and middle temporal gyrus, volumes of hippocampus, pallidum, corpus callosum and inferior lateral ventricle (Fig. 1A). In the aMCI-based model, some of the most important variables were volumes of hippocampus, amygdala and inferior lateral ventricle, thickness of entorhinal cortex, inferior temporal gyrus and fusiform gyrus (Fig. 1B). The atrophy patterns in both groups were similar, but not identical, sharing 3 out of 6 variables with highest loading. Comparing our variable loadings with the previous study [12], which combined over 1000 individuals from two multicentric studies, AddNeuroMed [68] and Alzheimer’s Disease Neuroimaging Initiative (ADNI; .loni.usc.edu/), we found variable loadings in all utilized datasets (AddNeuroMed, ADNI, combined) to be similar to our current models, particularly to the aMCI-based model, which shared 5 out of 6 variables with highest loading. The most important variables in the combined dataset were volumes of hippocampus, amygdala, and interior lateral ventricle, and thickness of entorhinal cortex, inferior and middle temporal gyrus. Although our current dataset comes from a single center in Sweden and is based on comparatively smaller number of participants (total 399 vs. 1074), similarity of the observed patterns of atrophy suggests they are stable across multiple populations in Europe and North America. Our models may therefore be well applicable to the data based on other populations.

We found further differences between the models when we applied them to predict progression from SCD to MCI or dementia. The dementia-based model achieved 100% specificity, but sensitivity was extremely low (10.6%). This finding makes this model partially less useful for clinical application unless the aim is to identify SCD patients with an extremely high risk of progression to MCI. In contrast, the aMCI-based model reached 72.3% sensitivity and moderate specificity of 60.9% in predicting progression from SCD to MCI. These finding suggests that the more advanced pattern of atrophy of patients used in training of dementia-based model identifies a small number of individuals at very high risk of clinical progression, while the milder yet developed atrophy pattern of aMCI patients results in superior sensitivity at the cost of specificity of the model. This suggests that models could be employed for different purposes. The AD dementia-based model could, for example, be utilized in identifying high-risk individuals for purpose of drug trial, while the aMCI model would be better used as a non-invasive population screening tool. However, comparing the *ROC AUC* directly between the models, the aMCI-based model was clearly superior to the AD dementia-based model, reaching 0.72 vs. 0.56 *AUC* (*P* = 0.037).

Though there are multiple studies using supervised learning and multivariate analysis to predict progression from MCI to dementia using structural MRI data [12, 20, 21, 69–72], there is limited number of studies attempting to predict progression from SCD to MCI [22, 73, 74].

Previously [22], we used OPLS to predict progression from SCD to MCI using a model trained on healthy controls and patients with probable AD dementia from the Australian Imaging Biomarkers and Lifestyle flagship study of ageing (AIBL). In line with our expectations, our aMCI-based model achieved lower specificity (60.9% vs. 95.4%) but a superior sensitivity (72.3% vs. 38.1%) to the previous model. The *ROC AUCs* could not be directly compared, as it was not reported in the previous study. Our dementia-based model, on the other hand, was more accurate in predicting clinical progression (100% vs. 95.4% specificity). It was however less sensitive than the previous model (10.6% vs. 38.1%). This was despite similar overall cognitive performance (mean MMSE 20.2 vs. 20.4) and *APOE* status (71.8% vs. 75.0% ε carriers) of AD dementia participants in both studies. Yet, the different results could partially be explained by larger percentage of *APOE* ε4 carriers in CN group in the AIBL cohort (46.0 vs. 15.4%).

Another recent study used support vector machines and multimodal data, including structural MRI data from FreeSurfer, to predict progression from SCD to MCI over 7 years period [73]. In comparison, MRI-based model in this study reached lower sensitivity (41.8%) and higher specificity (73.1%) than our aMCI-based model, while our dementia-based model was less sensitive and more specific. This study used a different approach, training the algorithm using longitudinal data of the evaluated SCD individuals.

Another study from the same group [74] used machine learning to create regression framework by combination of sparse coding and random forest to assess and predict cognitive performance in SCD and MCI individuals by predicting global cognition test scores change (i.e. MMSE and Montreal Cognitive Assessment) using structural MRI. Predicted values correlated with real scores with Pearson’s coefficients up to 0.35. These results are not directly comparable to our current results – global cognition scores are only roughly transferable to a clinical syndrome, and do not consider some important factors such as age and education of the patient.

Predicting progression from SCD to MCI or dementia is a task of high clinical significance. With upcoming availability of new treatment options [75], predicting progression from SCD to MCI or dementia will be crucial to effectively screen individuals in earliest stages of the disease to commence the treatment as soon as possible to achieve a maximum effect [4–6]. While currently there is a number of highly specific diagnostic methods available (i.e. CSF sampling and PET imaging), these are largely unsuitable for screening purposes due to their cost and invasiveness. Emerging blood-based biomarkers [1, 76] are yet to be integrated into the routine clinical practice. Structural MRI in conjunction with pattern atrophy analysis could therefore be employed in selection of patients at high risk of clinical progression for further diagnostic workup. We argue that for this purpose, the utilized model should be optimized to be highly sensitive while maintaining moderate specificity. Based on our results, we argue that models based on aMCI patients would be better suited for this task than the current models based on AD dementia patients. Using a training dataset based on biomarker-defined aMCI and CN individuals and by optimizing the model creation we can train our model to detect patterns of ‘early AD-related atrophy’ rather than ‘developed AD-related atrophy’.

One of principal strengths of this study was our dataset. For the model training we included β-amyloid positive aMCI, AD dementia and β-amyloid negative CN participants. Longitudinal data then consisted of SCD individuals with over 8 years of longitudinal monitoring. Further, we used a well-established method of multivariate data analysis – an OPLS generated “disease severity index”, that has been repeatedly proven an effective tool in predicting progression from SCD to MCI and from MCI to dementia [12, 20–22]. The processing of structural MRI data was performed using widely available automated software package (FreeSurfer 6.0), facilitating the application of our model on external datasets, and minimizing the risk of bias or human error in data processing.

### Limitations

This study also has limitations. Prediction models based on structural MRI, though achieving high specificity in predicting development of MCI and dementia, do not reflect the underlying pathology and therefore need to be used in combination with other methods that allow the assessment of amyloid or tau pathologies. While achieving moderate amounts of sensitivity and specificity, the current model still fails to identify significant portion of future progressors (~ 28%). Arguably, the model could be further improved by inclusion of segmentation of structures affected early in course of AD such as hippocampal subfields, transentorhinal and perirhinal cortex, anterolateral and posteromedial entorhinal cortex and basal forebrain nuclei [77], automated methods for segmentation of some [78, 79], but not all of these structures are publicly available. However, addition of further MRI processing steps would take away one of the major advantages of our current approach, that is a relative simplicity and reproducibility of MRI processing involved. Further, performance of our dementia-based model could be negatively affected by the fact that CSF biomarkers were not available in part of AD dementia group (*n* = 7; 17.95%). Another concern might be the reproducibility of our results. Since our data come from homogenous population from single center in Sweden, we cannot rule out the possibility that we are detecting a population specific pattern, that would not apply to other datasets. However, as discussed above, atrophy patterns we observed are very similar to the atrophy patterns observed in previous large multicenter studies assessing individuals across multiple populations across Europe and North America [12]. Therefore, we believe that the observed patterns are not specific to only our current population.

## Conclusions

In this study, we found that the prediction models based on brain atrophy patterns of individuals with milder levels of atrophy (i.e. aMCI) offer higher sensitivity and moderate specificity compared to standard dementia-based models for the prediction of clinical progression from SCD to MCI or dementia using structural MRI data. Thus, these models may offer superior clinical value and should be further refined and explored.

## Data Availability

Pseudonymized data will be shared by request from a qualified academic investigator for the sole purpose of replicating procedures and results presented in the article and as long as data transfer is in agreement with EU legislation on the general data protection regulation and decisions by the Swedish Ethical Review Authority and Region Skåne, which should be regulated in a material transfer agreement.

## Abbreviations

AD: Alzheimer’s disease
Aβ42: β-amyloid 42 peptide
ADAS-cog: Alzheimer’s Disease Assessment Scale-Cognitive subscale
AIBL: Australian Imaging Biomarkers and Lifestyle flagship study of ageing
aMCI: Amnestic mild cognitive impairment
ANCOVA: Analysis of covariance
ANOVA: Analysis of variance
AUC: Area under curve
CN: Cognitively normal
CI: Confidence interval
CSF: Cerebrospinal fluid
eTIV: Estimated total intracranial volume
GDS: Global deterioration scale
MCI: Mild cognitive impairment
MMSE: Mini mental state examination
MRI: Magnetic resonance imaging
NIPALS: Non-linear iterative partial least squares
OPLS: Orthogonal projection to latent structures
PET: Positron emission tomography
ROC: Receiver operating characteristic
SCD: Subjective cognitive decline
